# Perception of typical migraine images on the internet: Comparison between a metropolis and a smaller rural city in Germany

**DOI:** 10.1371/journal.pone.0290318

**Published:** 2023-08-18

**Authors:** Till Hamann, Ja Bin Hong, Kristin Sophie Lange, Lucas Hendrik Overeem, Paul Triller, Florian Rimmele, Tim Patrick Jürgens, Peter Kropp, Uwe Reuter, Bianca Raffaelli

**Affiliations:** 1 Department of Neurology, Headache Center North-East, Universitätsmedizin Rostock, Rostock, Germany; 2 Department of Neurology, Headache Center, Charité Universitätsmedizin Berlin, Berlin, Germany; 3 Doctoral Program, International Graduate Program Medical Neurosciences, Humboldt Graduate School, Berlin, Germany; 4 Department of Neurology, KMG Klinikum Güstrow, Güstrow, Germany; 5 Institute of Medical Psychology and Medical Sociology, Rostock University Medical Center, Rostock, Germany; 6 Universitätsmedizin Greifswald, Greifswald, Germany; 7 Clinician Scientist Program, Berlin Institute of Health (BIH) at Charité, Berlin, Germany; Xiangtan University, CHINA

## Abstract

The medial portrayal of migraine is often stereotypical and inaccurate but reflects how society perceives migraine. The discrepancy between others’ views and the reality of affected individuals may negatively affect access to treatment and the disease course of patients with migraine. This study aimed to investigate whether images presented in the media as typical migraine attacks are perceived as realistic and representative by migraine patients in Rostock, a smaller town in rural Germany, and compare the results to those from Berlin, a large metropolis. We performed an online survey in Rostock. Migraine patients were shown ten images of migraine attacks, which were among the most downloaded stock pictures on the internet under the search term "migraine". They rated on a scale of 0–100 to what extent the pictures were realistic for migraine attacks (realism score), representative of their own migraine (representation score), or the society’s view of migraine (society score). In addition, we compared our results with a recently published study from the metropolitan region of Berlin. A total of 174 migraine patients completed our survey. Mean (SD) realism, representation, and society scores were 59.9 (17.5), 56.7 (18.3), and 58.4 (17.1) respectively. Images of older patients were perceived as significantly more realistic and representative than those of younger patients (*P* < .001). Patients in Rostock (rural region) rated the images as significantly more realistic and representative than survey participants in Berlin (metropolis). Migraine patients in a rural region found typical migraine images only moderately realistic and representative but to a higher degree than their counterparts from a metropolis.

## Introduction

Migraine is one of the most frequent primary headache disorders and is associated with high levels of impairment in multiple facets of daily life [1–4]. Migraine affects about 20% of women and 8% of men worldwide and causes the highest number of years lived with disability in patients aged 15–49 years [5].

Despite the high prevalence and burden, patients experience significant levels of stigma [6, 7] that further impact daily life [8]. The stigma surrounding migraine may be rooted in the condition’s invisible nature, as it is often imperceptible to bystanders due to the lack of discernible outward signs, particularly in interictal periods, and the paucity of reliable diagnostic imaging or biomarkers to objectify its presence [9, 10]. As a result, patients face barriers to being believed and their pain often gets dismissed or trivialized [11]. Numerous studies have also shown that migraine patients are undertreated even in resource-rich settings [12–14]. The high female prevalence of migraine may contribute to stigma and undertreatment, as women’s pain is often underestimated and undertreated by physicians compared to men’s [15–17]. Reduced help seeking, possibly aggravated by internalized stigma, may also lead to inadequate medical care. In fact, internalized stigma in patients suffering from mental-health disorders has been shown to be associated with reduced help-seeking [18]. Compared to healthy controls, migraine patients were found to have significantly lower rates of seeking psychiatric help, despite having higher levels of psychiatric symptoms [19].

Stigma impacts the lives of migraine patients negatively on multiple levels. For example, it may compel patients to hide their condition from their workplace supervisors, causing them to miss out on potential workplace accommodations that may minimize triggers and reduce disability [20, 21]. If they do disclose their condition, they may come across coworkers who disapprove of their frequent sick leaves leading to feelings of isolation and low mood [21]. Even the medical establishment seems to neglect migraine in both research and treatment, as reflected by the disproportionate underfunding for headache research [22], and the relative lack of headache medicine specialists in some countries [23], and stigma may play a significant role in these trends.

Reversing social misconceptions and stigma about migraine requires educating the public about the true nature of its symptoms and resulting disability [24]. Visual communications are one of the most effective ways to disperse health information to a wider audience, and are being used increasingly in healthcare interventions [25]. The use of image-based health communications is a powerful tool for conveying complex information, but currently lacks guiding theories or research evidence [25–27]. Two studies [28, 29] exist that have examined images depicting migraine attacks and both suggest conventional photographic representations of migraine attacks are inadequate. Gvantseladze et al. [28] searched popular online image banks for images representing migraine attacks, and found that they were predominantly photos of ectomorph, white, and female adults, half of whom had their hands on both temporal regions at the same time. They propose that the disparity between common images used to depict migraine and actual presentations of migraine attacks may lead to further social stigmatization [28].

A recent study from Berlin showed that patients with migraine do not consider this prevalent photographic representations of migraine attacks realistic [29]. In this study, patients with migraine and healthcare professionals were shown commonly downloaded images representing migraine attacks and were asked to rate them on their realism and representativeness of their own migraine. Interestingly, portrayals of men and elderly models were deemed more realistic than those of younger and middle-aged, female models, which stays in contrast with the epidemiology of migraine in Germany [30]. The analyzed patient cohort in this cohort was part of a metropolitan population.

Epidemiological studies comparing migraine prevalence in urban and rural regions have found a lower prevalence of migraine in rural regions [31]. Inhabitants of large urban areas have different access to the media but also to medical information than people in rural regions [32]. Thus, perception of the medial representation of migraine may differ based on the urbanity of the place of residence. Access to specialty care may also be more limited in rural areas, leading to more patients being treated by general practitioners [33] and resulting in differences in their care when compared to urban areas. For example, studies have shown that especially chronic migraine patients treated by general practitioners often do not get referred to a specialist and tend to receive acute and preventive medication not in accordance with local and international guidelines [34, 35]. This may lead to generally lessened awareness of migraine, its characteristics and proper treatment in public discourse.

Here, we aimed to expand the findings from the Berlin study [29] by surveying patients with migraine living in the Rostock area. Rostock is an old Hanseatic city at the Baltic Sea. With its around 200.000 inhabitants and sparsely populated rural peripheries, it’s a regiopolitan region of the state of Mecklenburg-West Pomerania. Our objective was to assess how patients living in a smaller city in a rural region perceive such stereotypical pictures of migraine. Moreover, we intended to analyze the differences between Rostock and Berlin—a comparison between the capital of Germany (metropolitan area) and a sparsely populated area in east-Germany.

## Methods

### Study design

This is an anonymous web-based survey study performed on evasys.uni-rostock.de–the survey system of the University of Rostock. The survey was distributed via e-mail with a personalized, one-time link. We emailed all patients from the Headache Center of the University of Rostock (“Headache Center North East”) who received a diagnosis of migraine per International Classification of Headache Disorders–3 (ICHD-3) criteria [36] from January 2021 until July 2022. If the questionnaire was not completed after three weeks, we sent reminders for a maximum of four times.

After an electronic written consent form, the survey included questions about demographic, occupational, and migraine characteristics, as described in a previous publication [29]. For this study, we added a question about the place of residence and whether the patients perceive their place of residence to be more rural or urban.

We then presented participants with ten photos representing migraine attacks. Details of photo selection have been published elsewhere [29]. In short, these were the ten most often downloaded one-person portrayal under the search term “migraine” from the stock photo website Adobe Stock [37]. The participants were asked to rate each photo on a scale from 0 to 100 for the following categories:

1. extent to which the photo represents a realistic migraine attack (realism score)
2. extent to which the photo represents their own migraine attacks (representation score)
3. extent to which the photo represents the society’s view on migraine attacks (society score) (S1 Appendix).

A score of 0 meant “not realistic/representable at all” and a score of 100 meant “fully realistic/representable”. If the score ranged from 0 to 33, we rated it as low, between 34 and 66 as moderate, and between 67 and 100 as high.

The realism and representation scores were recorded in the same way in the Berlin study, while the society score represents a new, additional item peculiar to this survey. In the Berlin study, all patients with a diagnosis of migraine being treated at a tertiary headache center at Charité—Universitätsmedizin Berlin received invitations to participate in the survey via mail.

### Objectives and outcomes

Primary outcomes were the mean realism score, representation score, and society score for all pictures. Secondary outcomes were the mean scores for the following categories of photos: female models (n = 7) vs. male models (n = 3), unilateral pain posture (n = 6) vs. bilateral pain posture (n = 3), and); younger models (n = 5) vs. older models (n = 4) [29]. Images were classified into these categories based on the models’ physical characteristics, such as facial wrinkles and on unanimous agreement of all authors. It should be noted that the exact age of the models depicted in photographs was unknown. The division was therefore made into “older” and “younger”, whereby the younger models appeared to be around 30–35 years old and the older ones around 60 years or older. One photo could not be unanimously allocated to one posture or age category, and was excluded from analyses for those categories.

Furthermore, we compared the mean realism and representation scores between Rostock migraine patients (RM) and Berlin migraine patients (BM) for all pictures and each category. Data for BM was extracted from a previously published study [29].

### Statistical analysis

Demographic and anamnestic characteristics as well as the realism, representation, and society scores were summarized with descriptive statistics. We report absolute frequencies (%) for categorical variables and mean and standard deviation (SD) for continuous variables. We compared scores within the RM group using paired-sample t-tests, and between RM and BM using independent t-tests. Dependent and independent continuous variables were tested for normality using the Shapiro-Wilk test of normality.

In addition, we assessed the association between the primary outcome with sex, age, body-mass-index (BMI), the highest level of education (1 = in school, 2 = school finished without graduation, 3 = secondary school diploma, 4 = high school diploma, 5 = completed apprenticeship, 6 = technical baccalaureate, 7 = baccalaureate, 8 = university degree), and the perception of the residence being rural or urban (1 = urban, 2 = mixed, 3 = rural). For this, we performed multiple regression analyses for each of the primary outcomes. Linearity was assessed by partial regression plots and plots of studentized residuals against predicted values. Independence of observations was assessed using the Durbin-Watson statistic. Homoscedasticity was assessed by visual inspection of a plot of studentized residuals against unstandardized predicted values. Multicollinearity was assessed by tolerance (> 0.1) and variance inflation factor (< 10) values. We checked for outliers using Cook’s distance (< 1).

Using a two-way mixed ANOVA we examined whether concordance of sex between image and participant influenced how the images were rated. The participant’s sex was the between-subjects factor, and score for images with male vs. female models the within-subjects factor. Homogeneity of variances (p > .05) and covariances (p > .001) was assessed by Levene’s test of homogeneity of variances and Box’s M test.

A *P* value ≤ .05 after correction for multiple comparisons with the Bonferroni method was considered statistically significant.

### Ethics approval and consent to participate

The study was approved by the ethics committee of the Universitätsmedizin Rostock (A 2022–0036). Participants were required to provide electronic consent prior to completing the survey. The Berlin study [29] had been approved by the ethics committee of the Charité–Universitätsmedizin Berlin (EA1/213/20).

## Results

### Population

Between March and July 2022, of the 481 patients being treated at our headache clinic with the diagnosis of migraine, 437 with an e-mail address received the survey link via e-mail. Among those, 174 patients completed the survey (response rate: 40%).

The demographic characteristics of RM are shown in comparison to BM in Table 1. Body weight was higher in RM compared to BM (75.5, SD 16.9 kg vs. 69.4, SD 14.6 kg, *P* < .001). In addition, RM had a lower average level of education, a more homogenous ethnic background, and fewer family members with migraine.

**Table 1:** Demographic characteristics of the participants.

Characteristics	Rostock (n = 174)	Berlin (n = 366)
**Age (years)**	43.3 ±12.7	45.3 ± 12.7
**Female sex**	145 (83.3)	318 (86.6)
**Northern or Central European descent**	163 (93.7) ^a^	314 (85.6)
**Height (cm)**	169.3 ± 9.6	168.9 ± 8.1
**Weight (kg)**	75.5 ±16.9 ^a^	69.4 ± 14.6
**Highest level of education**		
University degree	54 (31.0) ^a^	154 (42.1)
High School diploma	26 (14.9) ^a^	63 (17.2)
Technical baccalaureate	14 (8.0) ^a^	29 (7.9)
Apprenticeship	26 (14.9) ^a^	60 (16.4)
Intermediate secondary school diploma	44 (25.3) ^a^	38 (10.4)
General secondary school diploma	6 (3.4) ^a^	4 (1.1)
Other	4 (2.3) ^a^	19 (4.9)
**Close friends or family members with migraine**	58 (43.3) ^a^	196 (53.4)

Values are given in mean ± SD or n (%). RM = patients in Rostock. BM = patients in Berlin.

^a^ Indicates P-value of P < .05 in the comparison between patients from Rostock vs. Berlin

When asked about their place of residence, 82 RM (47.1%) indicated that they live in an urban place, 50 (28.7%) in a rural area, and 42 (24.1%) in a mixed urban and rural area.

### Realism score in patients with migraine from Rostock

**Fig 1:**
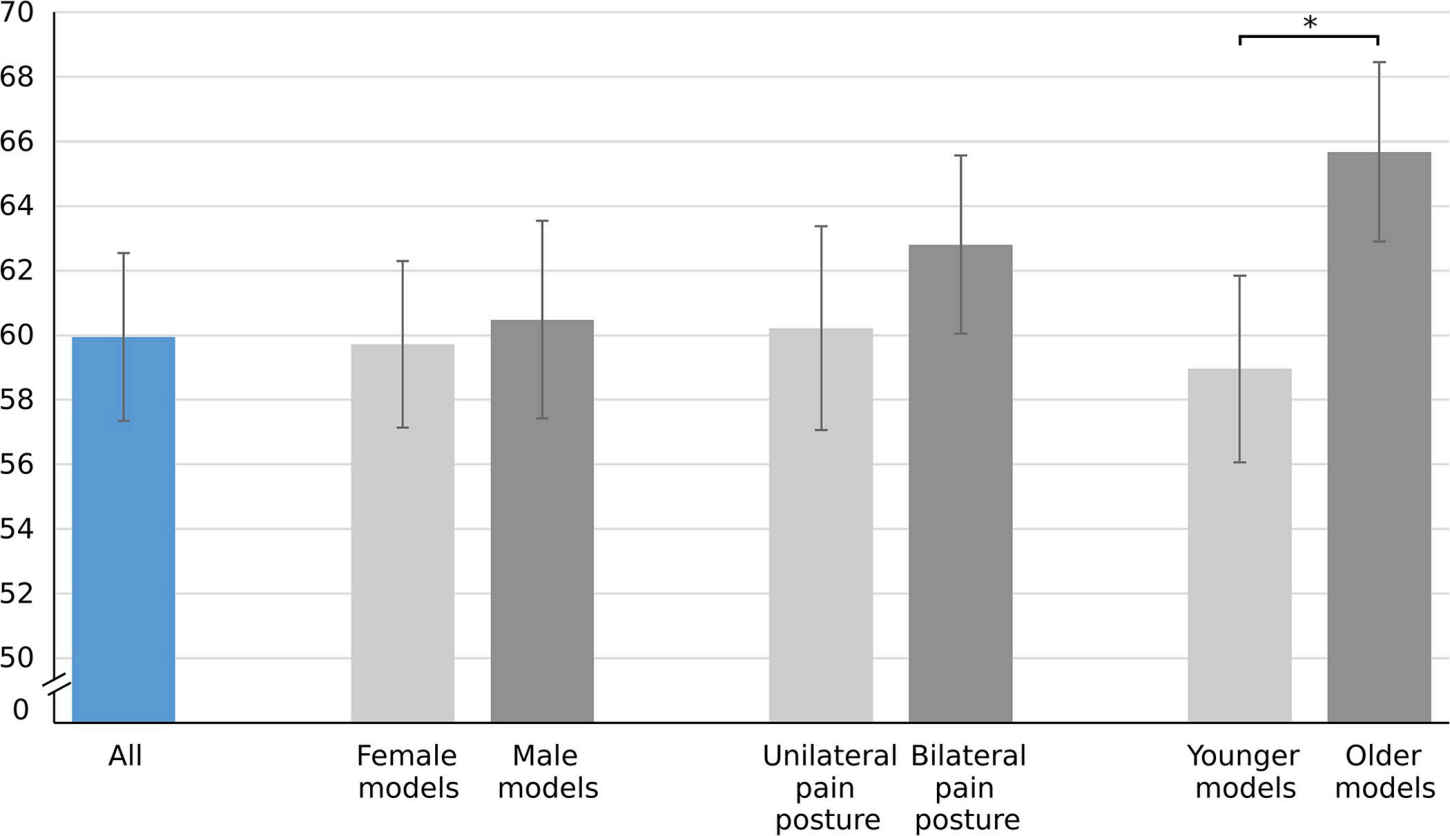
Mean realism scores for different image categories. The error bars represent 95% confidence intervals. * = statistically significant.

The mean realism score for all pictures was 59.9, SD 17.5. Photos with older models were perceived to be more realistic than those with younger ones (old: 65.7, SD 18.5 vs. young: 59.0, SD 19.5, *P* < .001). The differences between male and female models (male: 60.5, SD 20.6 vs. female: 59.7, SD 17.4, *P* = .42) and unilateral and bilateral pain postures (unilateral: 60.2, SD 21.2 vs. bilateral: 62.8 SD 18.5, *P* = .051) were not significant (Fig 1). There were no significant associations between the realism score and age, sex, education, and place of residence (S2 Table). S1 Table shows the mean scores for each individual picture.

### Representation score in patients with migraine from Rostock

**Fig 2:**
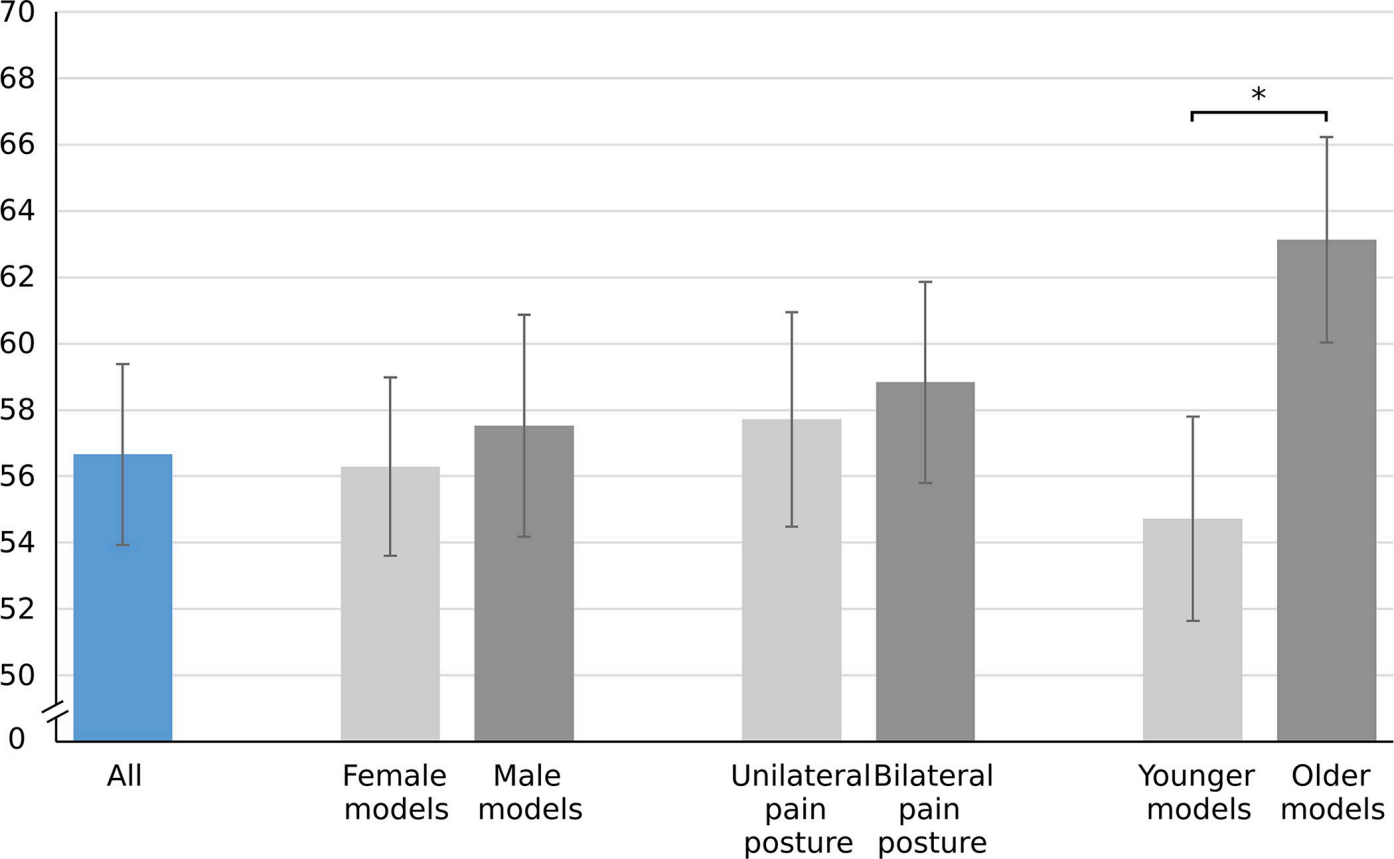
Mean representation scores for different image categories. The error bars represent 95% confidence intervals. * = statistically significant.

The mean representation score for the ten photos was 56.7 SD 18.3. Photos with older models were rated more representative than those with younger models (old: 63.1, SD 20.9 vs. young: 54.7, SD 20.7, *P* < .001, Fig 2). There was no significant difference between male and female models (male: 57.5, SD 22.5 vs. female: 56.3, SD 18.1, *P* = .27) and unilateral and bilateral pain postures (unilateral: 57.7, SD 21.8 vs. bilateral: 58.8, SD 20.4, *P* = .44). We did not detect any significant association between the representation score and the participant’s age, sex, education, or place of residence (S2 Table).

There was a statistically significant interaction between the participant’s sex and representation scores for images with female and male models *F*(1,170) = 5.60, *P* = 0.02, partial ƞ^2^ = 0.03. Images with male models were given significantly higher representation scores by male participants, when compared to those given female participants (mean difference [MD] 9.33, standard error [SE] 4.71, *P* = 0.05). Male participants gave significantly higher representation scores for images with male models than for images with female models (MD 7.21, SE 2.82, *P* = 0.02). There was no statistically significant difference in representation scores for images with female and male models given by female participants (MD -0.016, SE 1.20, *P* = .99).

### Society score in patients with migraine from Rostock

The mean society score for all photos was 59.7, SD 16.3. Male models, older models, and pictures with a bilateral pain posture reached significantly higher scores than female, younger and unilateral ones, respectively (Table 2). Of the demographic variables, age was negatively associated with the society score (β = -0.26, *P* = .002, S2 Table).

**Table 2:** Society scores for different image categories.

Criteria	Mean society score (SD)	Cohen’s d	*P*
**Female vs. male model**	57.1 (17.5) vs. 61.7 (18.9)	0.39	< .001
**Unilateral vs. bilateral pain posture**	56.2 (20.7) vs. 63.2 (17.4)	0.51	< .001
**Younger vs. older model**	58.0 (19.0) vs. 64.5 (18.1)	0.50	< .001

### Comparison between patients with migraine from Rostock and Berlin

In direct comparison, the photos reached higher representation and realism scores in RM than in BM (Table 3). This applied to all pictures as well as to each subgroup.

**Table 3:** Differences between mean realism and representation scores from RM and BM.

		RM	BM	Cohen’s d	*P*
**Mean realism Score (SD)**	All photos	59.9 (17.5)	48.8 (18.0)	0.63	< .001
	Female models	59.7 (17.4)	47.8 (18.3)	0.66	< .001
	Male models	60.5 (20.6)	51.0 (22.4)	0.43	< .001
	Unilateral pain posture	60.2 (21.2)	49.7 (21.0)	0.46	< .001
	Bilateral pain posture	62.8 (18.5)	51.8 (20.1)	0.56	< .001
	Younger models	59.0 (19.5)	47.7 (20.3)	0.56	< .001
	Older models	65.7 (18.7)	55.4 (20.9)	0.51	< .001
**Mean representation score (SD)**	All photos	56.7 (18.3)	44.4 (19.3)	0.64	< .001
	Female models	56.3 (18.1)	44.3 (19.8)	0.62	< .001
	Male models	57.5 (22.5)	44.8 (25.0)	0.52	< .001
	Unilateral pain posture	57.7 (21.8)	46.9 (26.0)	0.44	< .001
	Bilateral pain posture	58.8 (20.4)	46.0 (21.9)	0.60	< .001
	Younger models	54.7 (20.7)	43.2 (21.9)	0.54	< .001
	Older models	63.1 (20.9)	50.4 (22.7)	0.58	< .001

## Discussion

Our study shows that migraine patients living in a sparsely populated region perceive typical migraine pictures as only moderately realistic and representative of their migraine attacks. Pictures with older models were considered more realistic and representative than those with younger ones. In addition, participants classified older male models with bilateral pain postures as most representative of the society’s perception of migraine. In this cohort, the scores for realism and representation were significantly higher compared to previous study results in a metropolitan population. Furthermore, differences in the perception of images with male vs. female models seen in the Berlin cohort was not seen in the Rostock cohort.

Both mean realism and representation scores as well as the society score were under 60%. These are sobering results when considering that these ten pictures are the most frequently downloaded ones under the search term “migraine”. Especially images of the younger models or female models, who represent the main part of the migraine population [1], were given significantly lower society scores compared to their male and older counterparts.

Images depicting older models yielded significantly higher society, realism, and representation scores. It has been previously observed that expressions of pain in older faces lead to higher pain ratings compared to those of younger faces [38–40]. Participants in our study may have perceived the images with older models as having more pain, and thus as more realistic and representative of their own headache. Images of younger models could have been perceived as not capturing the intensity of a migraine attack. Additionally, although the incidence of migraine is highest in in young adulthood, migraine is most prevalent in adults aged 35–39 years [1], an age range that is typically no longer referred to as “young”, and the higher score given for older models may imply adults in that age range identify themselves more with older and elderly persons, rather than younger ones. Overall, these results are similar to the results from Raffaelli et al. [29], even though our study took place in a different setting.

The fact that pain reported by males is considered more believable and severe has been an often-observed phenomenon within gender bias in medicine [41, 42], leading to systematic underestimation and undertreatment of women’s pain [16]. In the study by Raffaelli et al. [29] images with female models yielded significantly lower realism scores, while in the present study, this gender difference was observed for the society score. It is paradoxical that images with male models are perceived to be more representative of society’s views on migraine attacks, when considering that the majority of individuals with migraine are women. It may imply an entrenched bias in the judgment of other’s pain that disproportionately minimizes pain suffered by women.

In the Rostock cohort, there was no significant difference between realism and representation scores of images with male and female models, in contrast to the results of the Berlin study, where images with male models were seen as more realistic. One hypothesis that could explain this difference is the cultural legacy of the socialist system of East Germany (Rostock being entirely part of former East Germany, and Berlin of former East and West Germany), where thirty years after reunification, women are still more likely to work full-time than in West Germany [43]. During the Socialist era in East Germany, more equal gender norms were instilled, while in West Germany, traditional norms with higher male earnings prevailed [44]. More equal gender norms may lead to less divergence in the perception of pain experienced by male and female models.

We also saw that male patients were more likely to give higher representation scores for images with male models, while the reverse was not true, that is, female patients were not more likely to give higher representation scores for images with female models. On the one hand, this might imply that male migraine patients feel even less well represented by the images of migraine that predominantly contain female models, while for female patients, the gender of the model showing a migraine attack is not as important. On the other hand, it might reflect a higher empathy of female participants for images of the opposite sex or their general habituation to untailored information materials, such as masculine images, in a male-dominated world. Exploring factors such as patient empathy, societal gender norms, and individual perceptions can provide valuable insights into addressing and improving the representation of both male and female migraine patients.

This is the very first study to assess the perception of migraine images by migraine patients in a small city with rural peripheries and compared the results to those from a metropolitan region. Our results reveal significant differences in the perception of migraine pictures depending on the place of residence: Patients from Rostock gave consistently higher realism and representation scores across all categories than the Berlin participants. Given that similar surveys with the same images were used in both studies, the reasons for this disparity are most likely associated with the inherent differences between the two cohorts. In addition to the contrasting level of urbanity, baseline characteristics of participants differed significantly. Rostock migraine patients have a lower average level of education, higher weight, fewer close contacts suffering from migraine, and a more ethnically homogeneous background.

Cross-sectional survey studies have shown that subjects with lower levels of education tend to trust health information and advertisements from television to a higher degree [45–47]. The tendency to trust medial representations shown to the general public may have led the participants in Rostock to accept the images shown more readily as an accurate portrayal of migraine.

Images that serve as illustrations for migraine may be used in mass media such as in newspaper or magazine articles, or in advertisements for products related to migraine. In the interest of attractiveness and overall appeal, creators of such material may prefer using images that depict models of a certain age and body shape. Previous studies showed that the majority of images of the typical migraine attack show a slim white female with a bilateral pain posture [28]. Our study shows that such pictures are not perceived as realistic or representative by migraine patients in a less urban area of Germany, similar to how they were perceived in a large metropolitan region [29], with differences that may be explained by factors such as level of education and require further exploration. The characterization of a typical migraine patient as a young, otherwise healthy female suffering from mild headache might even minimize the extent of disability and diverse symptoms that migraine causes [29, 48]. Portraying migraine attacks using such images may reinforce negative stereotypes about people with migraines, suggesting migraine sufferers are mentally weak and unable to cope with normal pressures and stress of daily life. Our study shows that certain populations more readily accept potentially stigmatizing images as realistic and representative and highlights a need to avoid such medial representations especially in more susceptible populations. One alternative could be to select images that show the full breadth and diversity of symptoms that migraine sufferers have to deal with, such as nausea and vomiting, sensitivity to light and noise, and severe headaches resulting in significant disability [48]. It is uncertain however, to what extent the use of such images in public media can help in destigmatizing migraine.

Misrepresentation and stigma surrounding an illness negatively influence the course of disease. Stigma represents a significant barrier to seeking help, reporting symptoms, and adhering to treatment recommendations [49]. Migraine patients report similar levels of stigma to patients with other disabling chronic illnesses such as epilepsy [7], preventing them from receiving adequate treatment [19]. Campaigns aimed at reducing stigma and educating the workforce on the nature of migraine disorders led to decreased indirect and total costs of migraine [50–52], some citing a return on investment as high as 490% [51]. Proper education that provides an accurate understanding of migraine has the potential not only to positively impact the lives of millions of sufferers but also to increase productivity and reduce migraine-related disability in young adults of working age. Adequate public education campaigns could start by using accurate, realistic portrayals of patients with migraine. Our results suggest that accurate depictions in media may be particularly important depending on the place of residence. An accurate representation of migraine in the media, especially in regions where the population is more vulnerable to its influences, could lead to a better understanding of migraine by both patients and the general public and encourage patients to seek adequate treatment.

One limitation of this study is the anonymity of the online-based survey. The lack of supervision could have led to less reliable data. We also informed our patients that the results of the survey would be compared against those from Berlin. This could have led to participants wanting to perform better in comparison and being more lenient in scoring the photos. In addition, the survey was only administered to migraine patients, precluding a comparison between views of affected and non-affected individuals, which may have provided additional insights.

Another limitation could stem from the fact that study participants were all patients being treated at a tertiary headache clinic, which may not adequately reflect the perception of common migraine images in all migraine sufferers living in rural areas. Patients referred to tertiary headache centers are similar in that they are more likely to have chronic migraine, a longer disease duration and larger disease burden compared to those who remain under the care of the general practitioner [53]. Comparing patients of two different tertiary headache centers may not provide sufficient contrast to draw meaningful conclusions about differences attributable to rurality.

To conclude, migraine patients from a small city in rural Germany perceived typical migraine images to be only moderately realistic and representative, albeit with higher ratings than those given by migraine patients from a large metropolitan area. These results highlight the need for more accurate portrayals of migraine in mass media, perhaps more so in less urban regions, where patients may be more susceptible to medial representations. More studies are needed to determine ways of visually representing migraine that reduce the stigma associated with the condition.

## Supporting information

S1 Checklist STROBE statement—checklist of items that should be included in reports of observational studies. (DOCX)

S1 Table Mean realism, representation, and society scores, as well as features for each image in the questionnaire. (DOCX)

S2 Table Multiple regression results for realism, society and representation scores. Multiple regression analyses for dependent variables realism, society and representation scores, with age, sex, BMI, degree of rurality and highest level of education entered into the model as independent variables.
(DOCX)

S1 Appendix Example questionnaire item (English translation). (DOCX)

S1 Data (CSV)
