# Supplementary material for: Perception of typical migraine images on the internet: Comparison between a metropolis and a smaller rural city in Germany
Source: PLoS One. 2023 Aug 18;18(8):e0290318. doi: 10.1371/journal.pone.0290318 (PMC10438019; doi:10.1371/journal.pone.0290318)
Supplement: S1 Table — (DOCX) [file pone.0290318.s002.docx]

**S1 Table.** **Mean realism, representation, and society scores, as well as features for each image in the questionnaire.**

| Item No. | Features | Mean realism score (SD) | Mean representation score (SD) | Mean society score (SD) |
| --- | --- | --- | --- | --- |
| 1 | female, bilateral, young | 60.92, SD 27.48 | 70.80, SD 24.00 | 53.51, SD 29.97 |
| 2 | female, unilateral, young | 60.00, SD 28.18 | 55.92, SD 26.99 | 54.94, SD 30.75 |
| 3 | male, bilateral, young | 52.07, SD 28.88 | 55.40, SD 26.15 | 48.28, SD 31.04 |
| 4 | female, unilateral, old | 55.69, SD 25.75 | 55.23, SD 27.34 | 55.40, SD 26.15 |
| 5 | female^a^ | 41.95, SD 29.62 | 36.32, SD 29.60 | 52.07, SD 28.88 |
| 6 | male, bilateral, old | 74.71, SD 23.82 | 73.74, SD 26.68 | 55.69, SD 25.75 |
| 7 | female, bilateral, old | 77.64, SD 23.91 | 73.33, SD 27.65 | 48.28, SD 31.04 |
| 8 | female, bilateral, young | 56.84, SD 30.64 | 50.17, SD 29.79 | 52.59, SD 32.41 |
| 9 | male, bilateral, old | 54.66, SD 26.50 | 55.86, SD 25.06 | 52.18, SD 28.44 |
| 10 | female, unilateral, young | 64.94, SD 26.18 | 57.59, SD 24.59 | 64.31, SD 27.06 |

SD = standard deviation. ^a^ Image 5 was not included in the analyses by age and laterality categories due to ambiguity in the two features.
