# Supplementary material for: Perception of typical migraine images on the internet: Comparison between a metropolis and a smaller rural city in Germany
Source: PLoS One. 2023 Aug 18;18(8):e0290318. doi: 10.1371/journal.pone.0290318 (PMC10438019; doi:10.1371/journal.pone.0290318)
Supplement: S2 Table — Multiple regression analyses for dependent variables realism, society and representation scores, with age, sex, BMI, degree of rurality and highest level of education entered into the model as independent variables. (DOCX) [file pone.0290318.s003.docx]

**S2 Table. Multiple regression results for realism, society and representation scores.**

|  | *B* | 95% CI for B  LL UL | | *SE B* | β | *P*-value |
| --- | --- | --- | --- | --- | --- | --- |
| Realism score, model *R^2^ = 0.025, ΔR^2^ = -0.006* | | | | | | |
| Constant | 59.59 | 37.26 | 81.91 | 11.30 |  | <0.001 |
| Age | -0.063 | -0.29 | 0.16 | 0.11 | -0.046 | .58 |
| Sex (female) | -4.79 | -12.23 | 2.65 | 3.77 | -0.099 | .21 |
| BMI | 0.30 | -0.26 | 0.86 | 0.28 | 0.082 | .30 |
| Degree of rurality | -1.27 | -4.61 | 2.06 | 1.69 | -0.063 | .45 |
| Highest level of education | 0.21 | -1.42 | 1.83 | 0.82 | 0.020 | .80 |
| Society score, model *R^2^ = 0.095, ΔR^2^ = 0.067* | | | | | | |
| Constant | 55.90 | 35.06 | 76.75 | 10.56 |  | <0.001 |
| Age | -0.34 | -0.55 | -0.13 | 0.11 | **-0.26** | **.002^a^** |
| Sex (female) | 1.47 | -5.48 | 8.42 | 3.52 | 0.032 | .68 |
| BMI | 0.40 | -0.12 | 0.93 | 0.27 | 0.12 | 0.13 |
| Degree of rurality | -1.02 | -4.13 | 2.10 | 1.58 | -0.052 | .52 |
| Highest level of education | 1.14 | -0.38 | 2.66 | 0.77 | 0.11 | .14 |
| Representation score, model *R^2^ = 0.012, ΔR^2^ =-0.019* | | | | | | |
| Constant | 68.25 | 44.27 | 92.24 | 12.15 |  | <0.001 |
| Age | 0.028 | -0.21 | 0.27 | 0.12 | <0.001 | .82 |
| Sex (female) | -4.05 | -12.04 | 3.94 | 4.05 | -0.079 | .32 |
| BMI | -0.14 | -0.73 | 0.47 | 0.31 | -0.036 | .65 |
| Degree of rurality | -1.17 | -4.75 | 2.42 | 1.82 | -0.054 | .52 |
| Highest level of education | -0.60 | -2.35 | 1.15 | 0.89 | -0.055 | .50 |

Multiple regression analyses for dependent variables realism, society and representation scores, with age, sex, BMI, degree of rurality and highest level of education entered into the model as independent variables. *B* = unstandardized regression coefficient; CI = confidence interval; LL = lower limit; UL = upper limit; *SE B* = standard error of the coefficient; β = standardized coefficient; *R^2^* = coefficient of determination; *ΔR^2^* = adjusted R2; BMI = body-mass-index (kg/m^2^).

^a^ = p < .05
