# Supplementary material for: Perception of typical migraine images on the internet: Comparison between a metropolis and a smaller rural city in Germany
Source: PLoS One. 2023 Aug 18;18(8):e0290318. doi: 10.1371/journal.pone.0290318 (PMC10438019; doi:10.1371/journal.pone.0290318)

**S3 Appendix.** **Example questionnaire item (English translation)**

On this and following pages, you will see ten pictures of migraine attacks. These are agency pictures posed with models. Please give your assessment of these ten images by moving the sliders.

Link to image: https://stock.adobe.com/de/images/portrait-of-stressed-young-housewife-in-modern-kitchen/53441576

How closely does this picture correspond to a realistic migraine attack?

0 = not at all

100 = completely


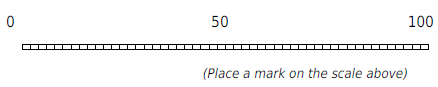


How closely does this image correspond to society's perception of a migraine attack?

0 = not at all

100 = completely


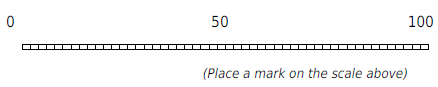


How closely does this image match your own migraines?

0 = not at all

100 = completely


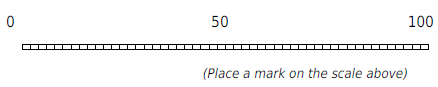

Supplement: S1 Appendix — (DOCX) [file pone.0290318.s004.docx]
